# Association of Genetic Variants at the *CDKN1B* and *CCND2* Loci Encoding p27*^Kip1^* and Cyclin D2 Cell Cycle Regulators with Susceptibility and Clinical Course of Chronic Lymphocytic Leukemia

**DOI:** 10.3390/ijms252111705

**Published:** 2024-10-31

**Authors:** Lidia Ciszak, Agata Kosmaczewska, Edyta Pawlak, Irena Frydecka, Aleksandra Szteblich, Dariusz Wołowiec

**Affiliations:** 1 Department of Experimental Therapy, Hirszfeld Institute of Immunology and Experimental Therapy, Polish Academy of Sciences, 53-114 Wroclaw, Poland; lidia.ciszak@hirszfeld.pl (L.C.); edyta.pawlak@hirszfeld.pl (E.P.); irena.frydecka@hirszfeld.pl (I.F.); aleksandra.szteblich@hirszfeld.pl (A.S.); 2 Clinical Department of Hematology, Cell Therapies and Internal Diseases, Wroclaw Medical University, 50-367 Wroclaw, Poland; dariusz.wolowiec@umw.edu.pl

**Keywords:** chronic lymphocytic leukemia (CLL), *CDKN1B* gene polymorphism, *CCND2* gene polymorphism, p27*^Kip1^* protein, cyclin D2

## Abstract

Beyond the essential role of p27*^Kip1^* and cyclin D2 in cell cycle progression, they are also shown to confer an anti-apoptotic function in peripheral blood (PB) lymphocytes. Although the aberrant longevity and expression of p27*^Kip1^* and cyclin D2 in leukemic cells is well documented, the exact mechanisms responsible for this phenomenon have yet to be elucidated. This study was undertaken to determine the associations between polymorphisms in the *CDKN1B* and *CCND2* genes (encoding p27*^Kip1^* and cyclin D2, respectively) and susceptibility to chronic lymphocytic leukemia (CLL), as well as their influence on the expression of both cell cycle regulators in PB leukemic B cells and non-malignant T cells from untreated CLL patients divided according to the genetic determinants studied. Three *CDKN1B* single-nucleotide polymorphisms (SNPs), rs36228499, rs34330, and rs2066827, and three *CCND2* SNPs, rs3217933, rs3217901, and rs3217810, were genotyped using a real-time PCR system. The expression of p27*^Kip1^* and cyclin D2 proteins in both leukemic B cells and non-malignant T cells was determined using flow cytometry. We found that the rs36228499A and rs34330T alleles in *CDKN1B* and the rs3217810T allele in the *CCND2* gene were more frequent in patients and were associated with increased CLL risk. Moreover, we observed that patients possessing the *CCND2*rs3217901G allele had lower susceptibility to CLL (most pronounced in the AG genotype). We also noticed that the presence of the *CDKN1B*rs36228499CC, *CDKN1B*rs34330CC, *CDKN1B*rs2066827TT, and *CCND2*rs3217901AG genotypes shortened the time to CLL progression. Statistically significant functional relationships were limited to T cells and assigned to *CDKN1B* polymorphic variants; carriers of the polymorphisms rs34330CC and rs36228499CC (determining the aggressive course of CLL) expressed a decrease in p27*^Kip1^* and cyclin D2 levels, respectively. We indicate for the first time that genetic variants at the *CDKN1B* and *CCND2* loci may be considered as a potentially low-penetrating risk factor for CLL and determining the clinical outcome.

## 1. Introduction

Chronic lymphocytic leukemia (CLL) has the highest prevalence of all adult leukemia cases in Western Europe and North America [1]. The incidence of CLL increases with age, with the median age at diagnosis being 70, and it is higher in those with a family history [2]. Clinically, patients often present with asymptomatic peripheral blood lymphocytosis or leukocytosis, lymphadenopathy, hepatosplenomegaly, bone marrow failure, and recurrent infections, and they often experience autoimmune hemolytic anemia or autoimmune thrombocytopenia [1,3,4]. Based on immunophenotypic studies, CLL is described as a progressive accumulation of monoclonal B lymphocytes with positive expression of CD5, CD19, and CD23 antigens in the peripheral blood, bone marrow, lymph nodes, and extra-nodal organs [1,3,4,5]. For many years, the accumulation of monoclonal B lymphocytes was thought to be due to a defect in programmed cell death rather than increased proliferation. Currently, based on in vivo measurements of CLL cell kinetics, it has been suggested that the CLL cell population may also include proliferating cells derived from the bone marrow, lymph nodes, or spleen [6,7]. Therefore, CLL is considered as a disease of both proliferation and accumulation of long-lived CLL cell clones, and CLL patients with higher birth rates are much more likely to exhibit active disease or develop progressive disease than those with lower birth rates [6,7,8].

Despite a remarkable phenotypic and cytological homogeneity, the disease exhibits a highly variable clinical presentation and evolution related to different prognostic factors [9,10]. It has been well documented that the aggressive disease course is associated with unmutated immunoglobulin heavy variable genes (IgVH) [1,11]; mutations in the *TP53* [1,12,13], *NOTCH1* [14,15,16], *BIRC3* [17], *SF3B1* [18], and *ATM* [19] genes; high expression of CD38 [20], the ξ-chain-associated protein 70 kDa [21], or CD49d [22]; elevated serum levels of β-2 microglobulin, thymidine kinase [23], or chemokines CCL3 and CCL4 [24,25]; and chromosomal aberrations such as deletion of the short arm of the p13 region of chromosome 17 (del17p) or del11q [19]. Studies performed by us and others indicate that CLL cells are characterized by increased expression of the p27*^Kip1^* protein, also known as cyclin-dependent kinase inhibitor 1B (CDKN1B) [26,27,28,29,30]. Unlike the majority of solid tumors, in which high expression of p27*^Kip1^* protein is linked with a favorable prognosis, the increased expression of this protein in CLL cells is associated with a more aggressive clinical course [28,30,31]. The p27*^Kip1^*protein was initially described as an inhibitor of the majority of known cyclin-dependent kinase (cdk)–cyclin complexes that play a crucial role in the cell cycle progression of all mammalian cells regulating the orderly transition from the G0 to G1, G1 to S, and G2 to M phases [32,33,34,35]. Recently, in addition to its canonical function as an anti-proliferative factor that prevents excessive and uncontrolled cell growth, many other functions of p27*^Kip1^* have been described. By interacting with proteins other than cdk–cyclin complexes, the p27*^Kip1^* protein participates in apoptosis, cytoskeleton modulation, cell motility, cell adhesion, autophagy, controlling DNA replication, gene transcription, and cell differentiation [36,37,38]. Although increased expression of the p27*^Kip1^* protein in leukemic cells is well documented, the exact mechanisms responsible for this phenomenon have yet to be elucidated. Similarly to the p27*^Kip1^* protein, increased expression of cyclin D2 was found in leukemic cells [27,29,39,40,41]. Cyclin D2 is a member of the family of D-type cyclins involved in the regulation of B lymphocytes passing through the G1 phase restriction point [42]. Literature data indicating differences between CLL cells and normal B lymphocytes in the regulation of early cell cycle progression underscore the special role of cyclin D2 in malignant B cells [39,43], especially in increasing the longevity of these cells in vivo [41].

Recently, much attention has been paid to the assessment of the relationships between genetic variants of the *CDKN1B* and *CCND2* genes (encoding p27*^Kip1^* and cyclin D2, respectively) and both their protein product levels and disease susceptibility. Thus, a number of single-nucleotide polymorphisms (SNPs) of the *CDKN1B* gene located within the 12p13.1-p12 chromosome encoding the p27*^Kip1^* protein have been investigated and found to be related to either regulation of p27*^Kip1^* protein expression or susceptibility to various cancers [44,45,46,47,48,49,50,51,52,53,54]. In particular, some, such as the p27V109G polymorphism, affect the stability of p27*^Kip1^* and diminish its intracellular content [49,55]. Similar to the p27*^Kip1^* protein, a number of SNPs of the *CCND2* gene located on chromosome 12p13.32 encoding cyclin D2 have been studied and found to be associated with the regulation of cyclin D2 protein expression and susceptibility to cancers [56,57,58,59,60,61]. In contrast to the well-documented role of *CDKN1B* and *CCND2* in the pathogenesis of solid tumors, knowledge is still lacking about the polymorphisms in the *CDKN1B* and *CCND2* genes in patients with CLL.

Therefore, we have undertaken this study to investigate a possible relationship between genetic variants located in the *CDKN1B* and *CCND2* loci and the clinical course of CLL. Based on the literature and functional predictions, we selected three polymorphisms within the *CDKN1B* gene: c.326T>G, V109G (rs2066827), −79C>T (rs34330), and −838C>A (rs36228499), as well as three polymorphic variants of the *CCND2* gene: c.*3825T>C (rs3217933), g.4405389A>G (rs3217901), and g.4279105C>T (rs3217810). In addition, we set out to find if there is an association between the studied genetic variants and the expression of p27*^Kip1^* and cyclin D2 proteins. Since clonal proliferation of malignant B lymphocytes and their accumulation is accompanied by an expansion of aberrantly functioning T cells, we determined the expression of p27*^Kip1^* and cyclin D2 proteins in both malignant B cells and non-malignant T cells from the peripheral blood of untreated CLL patients.

## 2. Results

### 2.1. Patient Characteristics

The distribution of *CDKN1B* and *CCND2* polymorphisms and susceptibility to CLL were studied in a group of 286 patients with previously untreated CLL recruited at the Clinical Department of Hematology, Cell Therapies and Internal Diseases, Wroclaw Medical University, Poland. For each patient, the diagnosis was established according to generally accepted criteria including absolute peripheral blood lymphocytosis ≥ 5 × 10^9^/L and the co-expression of CD5, CD19, and CD23 antigens on malignant cells. The disease stages were determined according to the Rai and Binet classifications. A control group of 283 healthy subjects originating from the same geographic area as the CLL patients was recruited from the blood bank in Wroclaw, the Hirszfeld Institute of Immunology and Experimental Therapy, Polish Academy of Sciences, Wroclaw, Poland, and the Lower Silesian Oncology Center, Wroclaw, Poland.

An association of genetic variants at the *CDKN1B* and *CCND2* loci with clinical course of CLL were examined in a group of 47 CLL patients. The clinical and laboratory features of the patients with CLL are summarized in Table 1.

### 2.2. CDKN1B and CCND2 Polymorphism Distribution and Susceptibility to CLL

In the first step, we examined the distribution of the genotypes and alleles of the selected polymorphisms of the *CDKN1B* and *CCND2* genes in the CLL patients and control subjects, and we analyzed the relationship between the polymorphisms studied and susceptibility to CLL. A detailed analysis of the genotypes and allele distribution is shown in Table 2 and Table 3. No polymorphism data for the CLL patients and healthy subjects demonstrated deviations from HWE (*p* < 0.05).

Of the three polymorphisms of the *CDKN1B* gene, we observed significant differences in the genotype frequencies at two polymorphic sites, rs36228499 and rs34330, between the CLL patients and healthy controls (Table 2). For the rs36228499 polymorphic locus, we found a significantly higher frequency of the CA and AA genotypes among the CLL patients compared to the controls (46.2% vs. 43.1% and 24.1% vs. 16.6%, respectively), indicating that the presence of the A allele (CA and AA genotypes, the dominant model) increased the risk of disease by about 1.6-fold compared to homozygous CC (OR = 1.60, 95% CI: 1.13–2.26, *p* = 0.008). The rs34330 polymorphic site also appeared to be associated with susceptibility to CLL. Analysis of the allele distribution showed that the T allele significantly increased the risk of CLL (OR = 1.40, 95% CI: 1.02–1.91, *p* = 0.03). Moreover, individuals with the rs34330TT genotype were 2.5 times more susceptible to CLL than those with the rs34330CC genotype (OR = 2.54, 95% CI: 0.96–6.76, *p* = 0.05).

For the *CCND2* gene polymorphisms tested, two seemed to be associated with the risk of CLL: loci rs3217901 and rs3217810 (Table 3). We found that individuals with the rs3217901AG genotype had about 0.70 lower susceptibility to CLL compared to those with the rs3217901AA genotype (OR = 0.69, 95% CI: 0.48–0.99, *p* = 0.04). In addition, a trend toward decreased disease risk among patients possessing the G allele (GG and AG genotypes, the dominant model) compared to those who were homozygous AA was observed (OR = 0.72, 95% CI: 0.52–1.02, *p* = 0.08). For the rs3217810 polymorphic locus, analysis of the allele distribution showed that the T allele was significantly overrepresented in the CLL patients compared to the controls (14.2% vs. 9.5%). Moreover, the presence of the T allele (CT and TT genotypes, the dominant model) increased the risk of disease about 1.6-fold compared to homozygous CC (OR = 1.57, 95% CI: 1.05–2.35, *p* = 0.03).

The haplotype analysis showed the presence of all haplotypes with a frequency higher than 3% in both the CLL patients and the healthy subjects (Table 4). The global distribution of haplotypes differed significantly between the CLL patients and the healthy individuals (χ^2^ = 45.37, *p* = 0.0000008). As shown in Table 4, both *CDKN1B*rs36228499C/*CDKN1B*rs34330C/*CDKN1B*rs2066827G/*CCND2*rs3217933T/*CCND2*rs3217901A/*CCND2*rs3217810C and *CDKN1B*rs36228499A/*CDKN1B*rs34330C/*CDKN1B*rs2066827T/*CCND2*rs3217933T/*CCND2*rs3217901A/*CCND2*rs3217810C haplotypes were observed significantly more frequently in the patients with CLL than in the healthy individuals, which increased the risk of CLL by about 10.6-fold and 1.6-fold, respectively (OR = 10.64, 95% CI: 3.01–37.65, *p* = 0.000006 and OR = 1.65, 95% CI: 1.19–2.29, *p* = 0.0029, respectively). Moreover, haplotypes *CDKN1B*rs36228499C/*CDKN1B*rs34330C/*CDKN1B*rs2066827T/*CCND2*rs3217933T/*CCND2*rs3217901A/*CCND2*rs3217810C and *CDKN1B*rs36228499C/*CDKN1B*rs34330C/*CDKN1B*rs2066827T/*CCND2*rs3217933T/*CCND2*rs3217901G/*CCND2*rs3217810C were observed significantly less frequently in the patients with CLL than in the controls, which decreased the risk of CLL by about 0.6-fold and 0.5-fold, respectively (OR = 0.66, 95% CI: 0.49–0.89, *p* = 0.0062 and OR = 0.54, 95% CI: 0.34–0.87, *p* = 0.0098, respectively).

### 2.3. CDKN1B and CCND2 Gene Polymorphisms and CLL Outcome

Then, we asked whether the polymorphisms examined in this study affected the clinical course of the disease. Due to the problem of obtaining complete medical information for all CLL patients enrolled in the genetic assay, we were only able to analyze the association between *CDKN1B* and *CCND2* gene polymorphisms in relation to disease progression in 47 patients. A detailed analysis of genotype distribution in this patient cohort is shown in Table S1. Since only four patients exhibited the *CDKN1B*rs34330TT genotype and three patients had the *CDKN1B*rs2066827GG genotype (Table S1), we combined the individuals with the CT and TT genotypes and the individuals with the TG and GG genotypes into groups of patients possessing the T allele (T+ carriers) and the G allele (G+ carriers), respectively. Given that the presence of the A allele at the *CDKN1B*rs36228499 polymorphic locus increased the risk of CLL (the dominant model) (Table 2), we combined the individuals with the CA and AA genotypes into the group of patients carrying the A allele (A+ carriers). Next, we compared the Kaplan–Meier curves for patient survival without lymphocyte doubling, progression to a higher Rai stage, and lymph node and organ progression, as well as the appearance of the indications for cytostatic treatment plotted for the CLL patients divided according to the polymorphic variants of the genes tested.

**Figure 1:**
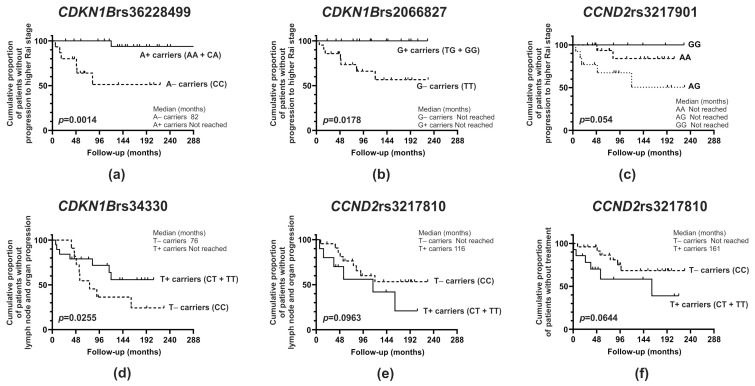
Cumulative probability of progression to a higher Rai stage, lymph node and organ progression, and treatment-free survival in CLL patients stratified according to the *CDKN1B* and *CCND2* gene polymorphisms. (**a**–**c**): Cumulative probability of progression to a higher Rai stage-free survival in CLL patients divided according to the genetic variants of the *CDKN1B*rs36228499 (**a**) and *CDKN1B*rs2066827 (**b**) polymorphic sites as well as the *CCND2*rs3217901 polymorphic locus (**c**). (**d**,**e**): Cumulative probability of lymph node-free survival in CLL patients stratified according to the genetic variants of the *CDKN1B*rs34330 (**d**) and *CCND2*rs3217810 (**e**) polymorphic sites. (**f**): Cumulative probability of treatment-free survival in CLL patients divided according to the genetic variants of the *CCND2*rs3217810 polymorphic locus. The *p*-value was obtained using the log rank test.

Although we determined no prognostic impact of the investigated *CDKN1B* polymorphisms on the survival of patients without lymphocyte doubling, a significant effect of *CDKN1B* polymorphic variants on progression to a higher Rai stage as well as lymph node and organ progression was observed (Figure 1, Table 5). As shown in Figure 1a,b, homozygous rs36228499CC and rs2066827TT displayed significantly shortened transition times to a higher Rai stage compared to A+ carriers and G+ carriers, respectively. Consistent with this, during the follow-up period, the frequency of homozygous rs36228499CC was markedly higher in patients with progression to a higher Rai stage, as six out of seven progressive patients were homozygous CC. In contrast, 9 out of 31 patients without progression to a higher Rai stage had the rs36228499CC genotype (χ^2^ = 7.68, *p* = 0.0096). As for the rs2066827 polymorphic locus, the frequency of homozygous TT was markedly higher in patients with progression to a higher Rai stage, as all patients (seven individuals) were homozygous TT. In contrast, 15 out of 30 patients without progression to a higher Rai stage had the rs2066827TT genotype (χ^2^ = 5.89, *p* = 0.0342). Moreover, as shown in Figure 1d, lymph node and organ progression occurred significantly earlier in homozygous rs34330CC compared to patients possessing the T allele (T+ carriers). Consistent with this, during the follow-up period, the frequency of homozygous rs34330CC was markedly higher in patients with lymph node and organ progression, as 9 out of 16 progressive patients were homozygous CC. In contrast, 3 out of 15 patients without lymph node and organ progression had the rs34330CC genotype (χ^2^ = 4.29, *p* = 0.05).

Regarding the *CCND2* gene polymorphism, since only two patients displayed the rs3217810TT genotype (Table S1), we combined those with the CT and TT genotypes into the group of patients carrying the T allele (T+ carriers). We found no prognostic impact of the investigated *CCND2* polymorphisms on patient survival without lymphocyte doubling. In contrast, we observed a significant influence of the rs3217901 polymorphic variants on progression to a higher Rai stage (Figure 1c) and an impact of the rs3217810 genetic variants on lymph node and organ progression as well as treatment implementation (Figure 1e,f, Table 5). It is noteworthy that heterozygous rs3217901AG had a significantly shortened time to progression to a higher Rai stage compared to homozygous AA and GG (Figure 1c). In line with this, the frequency of Rai stage progression during the follow-up period was non-significantly higher in heterozygous rs3217901AG than in AA and GG homozygotes, as five out of seven progressive patients had the rs3217901AG genotype. In contrast, 15 and 7 out of 30 patients without progression to a higher Rai stage were AA and GG homozygotes, respectively (χ^2^ = 5.44, *p* = 0.066). Furthermore, as shown in Figure 1e,f, a tendency toward earlier lymph node and organ progression as well as treatment onset was observed in carriers of the T allele (T+ carriers) at the rs3217810 polymorphic locus compared to homozygous CC carriers.

As a significant effect of all the studied polymorphic variants of the *CDKN1B* gene, as well as the *CCND2*rs3217901 and *CCND2*rs3217810 polymorphisms on CLL outcome, was observed, and a marked deviation from equilibrium of the *CCND2*rs3217933 polymorphism in the CLL patients was found (Table S1). We compared laboratory indicators, including those of prognostic significance such as IgVH mutated status (Table S2), LDH, and β2-microglobulin among the CLL patients stratified according to their polymorphic variants (Tables S3 and S4). Although there were no significant differences in these analyses, the trends toward lower white blood cell (WBC) counts in the G+ carriers of the *CDKN1B*rs2066827 polymorphic locus compared to homozygous TT (Table S3) and higher β2-microglobulin levels in heterozygous *CCND2*rs3217933TC in comparison with the carriers of the TT genotype were observed (Table S4).

In addition, we performed multivariate logistic regression analysis to stratify the influence of genetic and non-genetic factors on the risk of CLL progression shown by lymphocyte doubling, progression to a higher Rai stage, lymph node and organ progression, or cytostatic treatment implementation. We examined the clinical parameters, such as IgVH mutational status, WBC, lymphocyte and platelet counts, Hb, LDH, and β-2 microglobulin levels. We also assessed the predictive significance of the level of p27^Kip1^ and cyclin D2 in leukemic CD19+CD5+ cells and CD3+ cells, the fraction of apoptotic cells in the subset of CD19+ and CD3+ PB lymphocytes, and apoptotic CD19+ and CD3+ lymphocytes within PBMC. We found significant associations between the following clinical or immunological factors and CLL progression: IgVH mutated status (OR = 0.29, 95% CI: 0.09–0.99, *p* = 0.0487), WBC count (OR = 1.03, 95% CI: 1.01–1.06, *p* = 0.0054), lymphocyte count (OR = 1.04, 95% CI: 1.01–1.07, *p* = 0.0060), β2-microglobulin level (OR = 3.75, 95% CI: 1.09–12.9, *p* = 0.036), apoptotic fraction within CD19+ cells (OR = 0.76, 95% CI: 0.60–0.97, *p* = 0.0277), and the proportion of both apoptotic CD19+ and CD3+ cells within PBMCs (OR = 0.65, 95% CI: 0.44–0.96, *p* = 0.0315 and OR = 1.35, 95% CI: 1.003–1.81, *p* = 0.0481, respectively). In addition, among the genetic variants studied, logistic regression analysis identified the *CDKN1B*rs34330TT genotype and *CDKN1B*rs2066827T allele as predictors of CLL progression (OR = 0.02, 95% CI: 0.001–0.32, *p* = 0.0066 and OR = 0.05, 95% CI: 0.003–0.74, *p* = 0.03, respectively).

### 2.4. Functional Significance of CDKN1B and CCND2 Genetic Variants in CLL

**Figure 2:**
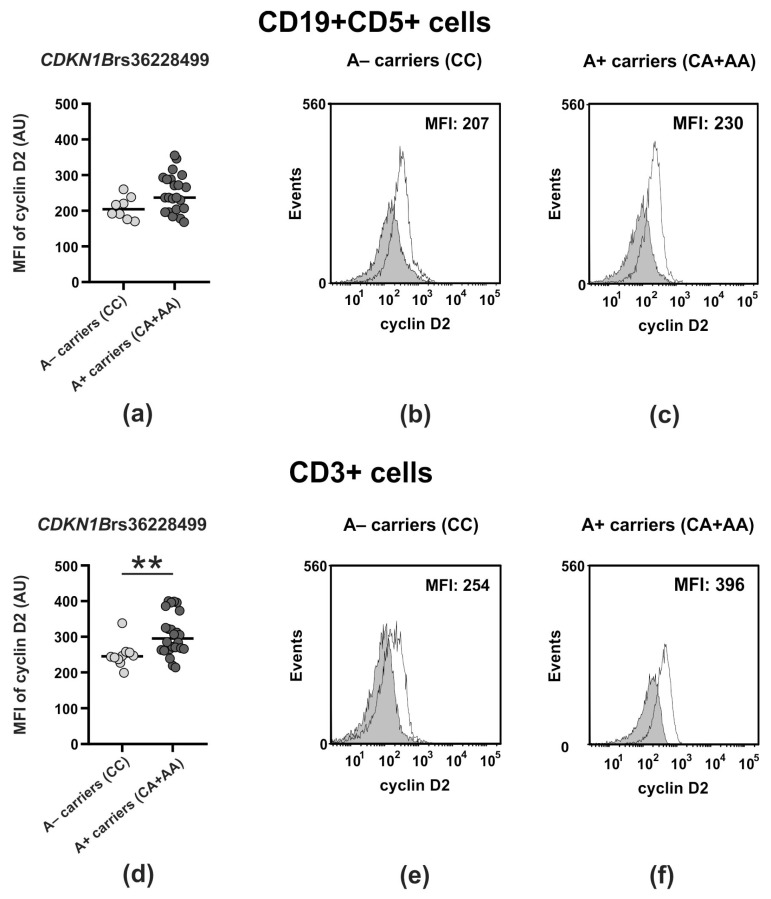
Association between genetic variants of the *CDKN1B*rs36228499 polymorphic locus and cyclin D2 expression in CLL patients. (**a**,**d**) The graphs show the mean fluorescence intensity (MFI) of cyclin D2 protein in PB CD19+CD5+ (**a**) and CD3+ (**d**) cells in A− and A+ carriers of the *CDKN1B*rs36228499 polymorphic site. The horizontal lines represent the median values. Differences between studied groups were evaluated using the Mann–Whitney U test. (**) signifies a statistically significant difference *p* < 0.01. (**b**,**c**,**e**,**f**): Cytometric analysis of cyclin D2 protein expression in CLL patients divided according to the genetic variants of the *CDKN1B*rs36228499 polymorphic locus. Histograms show cytometric analysis of cyclin D2 expression in PB CD19+CD5+ (**b**,**c**) and CD3+ (**e**,**f**) cells co-expressing cyclin D2 protein in A− and A+ carriers. PBMCs were gated using FSC/SSC profiles followed by gating on CD19+CD5+ (**b**,**c**) or CD3+ (**e**,**f**) to identify CD19+CD5+ and CD3+ cells for further analysis of cyclin D2 protein expression in PB CD19+CD5+ and CD3+ cells. Black line curves show cyclin D2-fluorescence of cells within PB CD19+CD5+ and CD3+ cells. Gray areas represent the isotype controls. The numbers located on the histograms represent the cyclin D2-dependent signal intensity (MFI).

Given the observed effects of the genetic variants of *CDKN1B* and *CCND2* on the different clinical courses of CLL, we asked whether these genetic variants affect the expression of the cell cycle regulators they encode: the p27^Kip1^ and cyclin D2 proteins, respectively. To answer this question, we performed functional studies determining the expression level of p27^Kip1^ and cyclin D2 proteins in both leukemic (CD19+CD5+) cells and non-malignant T cells, as well as the rate of apoptosis in CD19+ and CD3+ cells from the peripheral blood of CLL patients divided based on genotype at all polymorphic sites of the *CDKN1B* and *CCND2* genes examined. The results are summarized in Table 6 and Table S5 and illustrated in Figure 2, Figures S1 and S2.

With regard to the *CDKN1B* polymorphic variants, the only functional significance was noted in a subset of CD3+ lymphocytes and concerned the level of both p27^Kip1^ and cyclin D2. We found associations between the promoter polymorphism rs34330 and p27^Kip1^ expression, as well as between the rs36228499 polymorphic site and cyclin D2 levels. In detail, we observed that CLL patients carrying the rs34330 T allele (T+ carriers) expressed markedly higher levels of p27^Kip1^ protein in CD3+ cells compared to patients who did not possess this variant (Table 6, Figures S1 and S2g,h). For the rs36228499 polymorphic site, A+ carriers expressed significantly higher levels of cyclin D2 in CD3+ cells than A− carriers (homozygous CC) (Table 6, Figure 2); although we observed a similar association in a subset of leukemic CD19+CD5+ cells, it was only of marginal significance. Similarly, regarding the *CCND2* polymorphic variants (Table S5, Figure S1), we found non-significantly higher expression of cyclin D2 in leukemic CD19+CD5+ cells for A+ carriers at the rs3217901 polymorphic site (AG and AA genotypes) in comparison to A− carriers (homozygous GG).

We found no statistically significant differences in the rate of apoptosis in CD19+ as well as CD3+ cells between the groups of CLL patients stratified by genotype at all the studied polymorphic sites of the *CDKN1B* and *CCND2* genes (Table 6 and Table S5).

## 3. Discussion

Available data, including ours, have demonstrated that the ex vivo apoptotic rate of leukemic B and T cells may differ between CLL patients regarding the clinical stage of the disease, which points to a possible association with intrinsic signals affecting cell division [62]. To address this issue, we analyzed the associations of six polymorphic sites in the *CDKN1B* and *CCND2* genes for p27^Kip1^ and cyclin D2, respectively (G0/G1 phase regulators involved in cell proliferation and apoptosis) for CLL susceptibility and clinical course. We also examined the p27^Kip1^ and cyclin D2 protein levels in freshly isolated PB leukemic B and T cells with regard to the genetic determinants of the outcome of CLL. To our knowledge, this is the first study focused on the association of genetic variations of genes encoding p27^Kip1^ and cyclin D2 with CLL risk and clinical outcomes. We found that five out of six polymorphisms in the cell cycle pathway studied showed a significant risk for CLL, the highest risk being evident for the *CDKN1B*rs34330TT genotype (OR = 2.54). In the univariate analysis, we observed that the presence of the *CDKN1B*rs36228499A, *CDKN1B*rs34330T, *CDKN1B*rs2066827G, and *CCND2*rs3217810T alleles, as well as the *CCND2*rs3217901AA genotype, were individually predisposed to CLL in the Polish population. However, the *CDKN1B*rs2066827G allele and the *CCND2*rs3217901AA genotype were associated with CLL susceptibility with borderline significance. Yet, we did not find any associations between *CCND2*rs3217933 polymorphisms and susceptibility to CLL. Also, the haplotype analysis we performed showed that the presence of an individual risk allele, namely *CDKN1B*rs36228499A, *CDKN1B*rs2066827G, or *CCND2*rs3217810T, as a part of the haplotype predisposed to CLL could confer susceptibility by up 10-fold (namely the *CDKN1B*rs36228499C/*CDKN1B*rs34330C/*CDKN1B*rs2066827G/*CCND2*rs3217933T/*CCND2*rs3217901A/*CCND2*rs3217810C haplotype).

There is increasing evidence in CLL that ongoing birth and death within the CLL clone, as well as the number of leukemic B cells, is determined by an active interaction between these two processes. Our results showing high prevalence of the *CDKN1B*rs36228499A or *CDKN1B*rs2066827G alleles promoting p27^Kip1^ increase and cell accumulation in CLL patients are in line with those obtained for the protective action of both genetic variants in organ transplantation and tumors [49,63,64,65,66,67,68,69]. In particular, a significant prevalence of the *CDKN1B*rs36228499A allele was found to be associated with impeded risk of target vessel vascularization and restenosis in a transplanted organ and gave support for graft acceptance [68,69]. The *CDKN1B*rs2066827G allele was shown to be protective in solid tumors, as it was found to be less common in pancreatic, thyroid, and ovarian cancer patients compared to healthy controls [49,66,67]. Furthermore, patients with pancreatic cancer carrying the *CDKN1B*rs2066827G allele displayed longer overall survival after treatment [63]. It has been demonstrated that the *CDKN1B*rs36228499A and *CDKN1B*rs2066827G alleles are associated with an increase in the transcription activity of the *CDKN1B* gene promoter, thus supporting cell cycle arrest and inhibition in cell proliferation [64]. However, it has also been reported that lymphocyte proliferation may be an important factor contributing to tumor mass growth in CLL [7,8,70]. The high prevalence of the *CDKN1B*rs34330T or *CCND2*rs3217810T alleles found in our cohort of CLL patients appears to confirm the latter suggestion, as those polymorphisms are associated with a decrease in p27^Kip1^ or an increase in cyclin D2 gene induction [47,71,72,73], respectively, which could be indicative of improved cell proliferative potency in addition to apparent longevity in CLL. Consistently, each polymorphic site (*CDKN1B*rs34330T or *CCND2*rs3217810T) was found to be more common in neoplastic cells isolated from several solid tumors [47,51,52,54,59,71,72,74,75,76,77]. Of note, regarding the *CCND2*rs3217901AG polymorphic genotype, although we found it to protect against CLL, it was also shown to affect the CLL outcome, with it being marginally related to aggressive disease. Previous studies found a significant association between the *CCND2*rs3217901 genotype and colorectal, oral, or ovarian cancers, indicating that the *CCND2*rs3217901GG and *CCND2*rs3217901AG genotypes confer the risk of malignancies related to the uncontrolled proliferation of neoplastic cells [56,58,59,78]. Although associations between *CDKN1B*rs34330, *CDKN1B*rs2066827, *CCND2*rs3217810, and *CCND2*rs3217901 polymorphisms and susceptibility to a large number of malignancies have been demonstrated, the available data in the literature regarding this point have not been consistent [54,69,75,76,79,80,81,82,83].

Remarkably, we noted that the *CDKN1B* and *CCND2* gene polymorphisms examined in this study might affect the clinical course of CLL, which extends their significance beyond a contribution to disease susceptibility. To the best of our knowledge, our study is the first to analyze in detail the associations between the *CDKN1B* and *CCND2* gene polymorphic variants and the time to CLL progression reflected in lymph node and organ progression, treatment onset, or progression to a higher Rai stage. Of note, we found relationships between the genotypes non-predisposing to CLL, namely *CDKN1B*rs36228499CC, *CDKN1B*rs2066827TT, and *CDKN1B*rs34330CC, within the p27^Kip1^ pathway, as well as the *CCND2*rs3217901AG genotype and an aggressive course of CLL. Regarding this point, the only exception was the *CCND2*rs3217810T allele predisposing to CLL, which was marginally associated with earlier disease progression. The results of the Kaplan–Meier analysis demonstrated that the transition time to a higher Rai stage was shorter for patients possessing *CDKN1B*rs36228499CC or *CDKN1B*rs2066827TT, as well as the *CCND2*rs3217901AG genotype, compared with patients lacking these variants. It is well accepted that transition to a higher CLL clinical stage indicates an unfavorable prognosis. Likewise, patients with the *CDKN1B*rs34330CC genotype or *CCND2*rs3217810T allele carriers were more prone to earlier lymph node and organ progression, also described as a predictor of poor clinical outcome. Patients possessing the *CCND2*rs3217810T allele were also found to have shortened time to treatment onset. The majority of the *CDKN1B* and *CCND2* genotypes demonstrated by us as determining a progressive CLL course (e.g., *CDKN1B*rs36228499CC, *CDKN1B*rs2066827TT, *CCND2*rs3217901AG, *CCND2*rs3217810T) were previously described as predictors for the risk of uncontrolled proliferation in solid tumors [46,49,63,68,74], and their prevalence in aggressive CLL could be associated with the enhanced proliferative potential of the circulating lymphocytes. Of note, it has recently been reported that the higher proliferation rate in the cell compartment in CLL correlates with a greater ability to enter spontaneous apoptosis ex vivo and is attributed to disease progression [62]. Among the polymorphisms determining aggressive CLL, the *CDKN1B*rs34330CC genotype appears to be the only exception, as it is a genetic variant associated with enhanced *CDKN1B* promoter activity and protein levels, which also emphasizes the significant role of arrested lymphocyte accumulation in CLL progression [47]. Our findings regarding the relationships between non-predisposing to CLL genotypes within the p27^Kip1^ and cyclin D2 pathways and a more severe clinical course of CLL are consistent with previous observations of the genes affecting the clinical course of autoimmune disorders [84,85].

To explain whether the genetic variants of *CDKN1B* and *CCND2* found here to be associated with a different clinical course of CLL might affect the protein expression of these cell cycle regulators, we performed functional studies. We did not find any significant differences between the protein levels of p27^Kip1^ and/or cyclin D2 in patient cohorts divided based on genetic determinants of the outcome of CLL, except for the *CDKN1B*rs36228499CC and *CDKN1B*rs34330CC genotypes determining progressive CLL. When compared to the levels seen in patients possessing the *CDKN1B*rs36228499A or *CDKN1B*rs34330T alleles, the common functional characteristics of patients with the *CDKN1B*rs36228499CC and *CDKN1B*rs34330CC genotypes were a statistically significant decrease in cyclin p27^Kip1^ and D2 levels (*p* = 0.002 and *p* = 0.01, respectively). This effect was evident only for the T cell compartment, thus clearly indicating T cell proliferation attempts during CLL progression. The aberrant expression of G1 phase regulators in T cells from patients possessing the *CDKN1B* genotypes determining more advanced CLL is unique information, and it emphasizes a special role of the non-malignant T cell population in the biology of CLL. Also, a novel and interesting observation of this study was the effect of the rs36228499 SNP within the promoter region of the *CDKN1B* gene on the expression pattern of cyclin D2, a product of the *CCND2* gene. Interestingly, this impact was shown primarily in T lymphocytes. It is not surprising that we observed this cross effect, since both the *CDKN1B* and *CCND2* genes are located close to each other in the same chromosome 12 region (12p13.1-p12 and 12p13.32, respectively). Therefore, the impact of *CDKN1B* SNP on the protein product levels of the *CCND2* gene located next to the gene where the SNP lies is very probable. This phenomenon needs to be fully explained, since there are very few papers devoted to this marker [47,68,69,86,87]. So far, van Tiel et al. [69] showed that the *CDKN1B*rs36228499 polymorphism is associated with differences in basal p27^Kip1^ promoter activity, but there are no data analyzing its impact on the proteins encoded by other genes located in close proximity on the same chromosome.

Beyond the essential roles of p27^Kip1^ and cyclin D2 in cell cycle progression, they were also shown to confer an anti-apoptotic function in PB lymphocytes [28,41,88]. Therefore, the loss of p27^Kip1^ and cyclin D2 primarily in the T cell populations found in the functional assay might indicate that progression of CLL is associated with an increased turnover of T cells rather than B cells. In line with the above suggestion, our logistic regression analysis revealed the opposite predictive significance of B and T cell apoptosis; the rate of apoptosis of T cells increases with CLL progression and is associated with other clinically poor prognostic factors such as IgVH unmutated status, high WBC and lymphocyte counts, and increased β2-microglobulin levels. Based on the current literature, the higher rate of apoptosis of T cells in CLL may be explained by the fact that the proliferating cells were shown to be more prone to apoptosis, thus indicating a more aggressive disease [62]. Therefore, our study clearly suggests that in CLL, non-malignant T cells appear to be an important effector population involved in the p27^Kip1^ and cyclin D2 genetic determination of the clinical outcomes of CLL. Maintaining an appropriate T cell population size in the PB as relevant support for leukemic B cell growth is consistent with the recent demonstration that T cells in CLL could prevent apoptosis of malignant B cells [89]. On the other hand, given that failed apoptosis of PB B cells is predictive of more aggressive CLL, we may conclude with caution that the different rate of protection of B and T lymphocytes from apoptosis in CLL could promote clonal expansion of B cells, increasing with CLL progression. This could be confirmed by the higher prevalence of genotypes supporting cell proliferation activity among progressive patients enrolled in the current study, which emphasizes the complex nature of CLL pathogenesis.

Although a weakness of our study is that the relatively small size of the patient cohort inhibited our ability to make strong conclusions, noteworthy relationship was observed between polymorphisms of the *CDKN1B* and *CCND2* genes and CLL course. Our novel finding may have prospective clinical applications because it shows that some polymorphisms are likely to be linked with rapid progression of CLL. Therefore, they may be considered as negative prognostic factors of shorter progression-free survival. Those findings must be corroborated by future studies aimed at determining whether these polymorphisms have independent prognostic value or are related to other biological and/or clinical factors of known prognostic significance. Another direction of future investigation would be to determine whether CLL patients carrying these polymorphisms would benefit from the early start of the treatment in the absence of currently admitted criteria. Our findings do not allow us to draw conclusions regarding the best therapeutical option to be proposed to the patients. However, it seems advisable to devise a study aimed at determining whether polymorphisms of the *CDKN1B* and *CCND2* genes have an impact on the efficacy of the different regimens currently used in CLL (Bruton’s tyrosine kinase inhibitors, inhibitors of BCL2 protein, anti-CD20 monoclonal antibodies).

## 4. Materials and Methods

### 4.1. Patients

All the participants provided written informed consent after the purpose of this study was explained. This study design was approved by the local bioethical committee at the Medical University of Wroclaw, Poland, and is in accordance with the Declaration of Helsinki of 1975.

### 4.2. Genotyping Studies

#### 4.2.1. *CDKN1B* and *CCND2* Polymorphism Selection

SNPs were selected across preselected cancer-related genes, choosing tagSNPs using the LD TAG SNP selection platform on the website of the National Institutes of Environmental Health Sciences (NIEHS) for Europeans (http://snpinfo.niehs.nih.gov/snptag.htm, accessed on 1 September 2006). This uses a refined greedy algorithm originally implemented in the TAGster software (version 1.0) for LD tagSNP selection, where linkage disequilibrium (LD) was measured based on r^2^ or composite linkage disequilibrium (CLD) [90].

SNPs in the *CDKN1B* gene, rs36228499 and rs2066827, and in the *CCND2* gene, rs3217810, rs3217901 and rs3217933 were genotyped using TaqMan^®^SNP Genotyping Assays, while SNP rs34330 in the *CDKN1B* gene was genotyped using PCR-RFLP (see Supplementary Materials for expanded information on each SNP) [91,92].

#### 4.2.2. DNA Extraction and Genotyping

For genomic DNA and SNP analysis, whole blood from both CLL patients and healthy individuals was collected in tubes containing ethylene–diamine–tetra acetic acid (EDTA) (Catalog #02.1066.001, Sarstedt AG & Co. KG, Nűmbrecht, Germany) and stored at −80 °C until use. Genomic DNA was extracted using a Invisorb Spin Blood Midi Kit (Catalog #1031110200, Symbios, Straszyn, Poland) according to the manufacturer’s instructions, and the purity and concentration were assessed spectrophotometrically (Thermo Scientific™Nano Drop 2000/2000c Spectrophotometer, Thermo Fisher Scientific, Waltham, MA, USA).

### 4.3. Cytofluorometry Study

#### 4.3.1. PBMC Isolation

Peripheral blood mononuclear cells (PBMCs) from CLL patients were separated from heparinized freshly drawn peripheral venous blood via buoyant density-gradient centrifugation on Lymphoflot (Catalog #02.1065.001, Sarstedt Aktiengessellschaft & Co, Nűmbrecht, Germany) and washed three times in phosphate-buffered saline (PBS) (without Ca^2+^ and Mg^2+^).

The PBMCs were suspended in 95% fetal calf serum (Catalog #CA5-104, CytoGen GmbH, Sinn, Germany) containing 5% dimethylsulfoxide (DMSO, Catalog #D8418, Sigma-Aldrich, St. Gallen, Switzerland) and stored in liquid nitrogen until use.

#### 4.3.2. Immunostaining of p27^Kip1^ Protein and Cyclin D2 and Flow Cytometric Analysis

The expression of p27^Kip1^ and cyclin D2 proteins was studied in leukemic (CD19+CD5+) as well as non-malignant T cells from the CLL patients using a triple or double immunostaining method, respectively, which is described in detail in the Supplementary Materials [29,93].

#### 4.3.3. Determination of Apoptosis (Assessed as Mitochondrial Membrane Potential [ΔΨm Low] Changes)

Apoptosis of B (CD19+) and T (CD3+) lymphocytes from CLL patients was determined using chloromethyl-X-rosamine staining (catalog #M7512, MitoTracker Red CMXRos, Molecular Probes, Eugene, OR, USA) according to the manufacturer’s instructions, which is described in detail in the Supplementary Materials [94].

### 4.4. Statistical Analysis

Statistical analyses of the clinical data and laboratory findings were performed using the Statistica 7.1 package (TIBCO Software Inc., Palo Alto, CA, USA) and GraphPad Prism 8.01 (GraphPad Software, San Diego, CA, USA). All the patients were followed-up, and the time lag to the occurrence of at least one of the following events was recorded: doubling of the peripheral lymphocyte count compared to the initial value, progression to a higher Rai stage, lymph node and organ progression, and the appearance of the indications for cytostatic treatment according to the NCI-Sponsored Working Group recommendations. The follow-up period ranged from 1 to 228 (median 110.0) months. During this period, doubling of the lymphocyte count was found in 14 patients, disease progression to a higher Rai stage was found in 7 patients, lymph node and organ progression was found in 16 patients, and cytostatic treatment had to be started in 15 patients. The indications for this treatment were the appearance of at least one of the symptoms of disease activity (six patients), rapid progression of lymphadenopathy and/or splenomegaly (four patients), and the appearance of anemia (five patients).

The clinical parameters and genotype distributions of the investigated CDKN1B and CCND2 gene polymorphisms are presented as absolute numbers and percentages for frequencies. The genotype frequencies were compared with the expected values for the distribution of genotypes in Hardy–Weinberg equilibrium (HWE) using the chi-square test (χ^2^). For age or morphological and biochemical indicators, the mean values and standard deviation were calculated. For all other analyzed variables, the median values and 25th and 75th interquartile ranges (IQ ranges) were calculated. All collected data were examined for normal distribution and for the homogeneity of variances using the Shapiro–Wilk test and Levene’s test, respectively. Since the data did not have a normal distribution and/or had heterogeneous variances, the Mann–Whitney U test or one-way analysis of variance with Kruskal–Wallis ranks were used as non-parametric alternatives.

The cumulative probability of survival without lymphocyte doubling, survival without progression to a higher Rai stage, survival without lymph node and organ progression, and survival without the appearance of an event justifying cytostatic treatment was calculated according to the Kaplan–Meier method. As endpoints, doubling of the peripheral lymphocyte count, progression to a higher Rai stage, lymph node and organ progression, and beginning cytostatic treatment were used, respectively. The Kaplan–Meier curves were compared using the F Coxa test. The frequencies of the occurrence of each analyzed event during the whole follow-up period between different patient groups were compared using the chi-square test. When the expected frequency in at least one cell was <5, Fisher’s exact test was used. In all analyses, differences were considered significant when *p* ≤ 0.05.

The combined effects of genetic markers and non-genetic factors were analyzed using multivariate logistic regression analysis and forward logistic regression analysis using the stepwise method. The odds ratios (ORs) are presented with 95% confidence intervals (CIs). The model was selected based on goodness-of-fit criteria such as the determination coefficient, R^2^, Cox–Snell, and Akaike Information Criteria (AIC), where a greater R^2^ and a lower AIC value were indicators of the best statistical fit. The goodness of fit of the regression model was assessed using the Hosmer and Lemeshow test.

## Figures and Tables

**Table 1:** Clinical and laboratory characteristics of the enrolled CLL patients.

Parameters	CLL Patients (n = 47)	Frequency (%)
**Age in years**	72. 8 ± 9.46	
**Gender**		
Female	21	44.7
Male	26	55.3
**Rai stage**		
0	22	46.8
1	8	17.0
2	3	6.4
3	2	4.3
4	5	10.6
**Binet stage**		
A	35	74.5
B	5	10.6
C	2	4.3
**IgHV status**		
Unmutated	16	34.0
Mutated	23	48.9
Undetermined	8	17.1
**Disease progression** Yes/no		
Doubling of the lymphocyte count	14/33	29.8/70.2
Progression to a higher Rai stage	7/40	14.9/85.1
Lymph node and organ progression	16/31	34.0/66.0
Indications for cytostatic treatment	15/32	31.9/68.1
Follow-up period (months)	110.0 (1–228)	
**Morphological indicators**		
WBC count (1 × 10^9^/L)	48.28 ± 34.25	
Lymphocyte count (1 × 10^9^/L)	41.06 ± 32.05	
Hb level (g/dL)	12.94 ± 1.78	
Platelet count (1 × 10^9^/L)	151.3 ± 53.27	
**Biochemical indicators**		
LDH (U/L)	202.62 ± 72.31	
β2-microglobulin (mg/L)	3.35 ± 1.55	

WBC—white blood cells; LDH—lactate dehydrogenase; IgVH—immunoglobulin variable heavy chain gene. Quantitative variables are presented as numbers and percentages. For age and laboratory parameters, the mean values and standard deviations (SDs) are presented. For the follow-up period, the median value and range are presented.

**Table 2:** Genotype and allele frequencies of selected polymorphisms within the *CDKN1B* gene in CLL patients and the control group.

Polymorphic Site	CLL Patients n (%)	Controls n (%)	χ^2^ Test	OR (95%CI)	*p*-Value
***CDKN1B*** **rs36228499**					
CC	85 (29.7)	114 (40.3)	**χ^2^ = 8.78** ***p* = 0.0124**	Reference	
**CA**	132 (46.2)	122 (43.1)		**1.45 (1.00–2.11)**	**0.05**
**AA**	69 (24.1)	47 (16.6)		**1.97 (1.24–3.14)**	**0.004**
HWE	*p* = 0.2106	*p* = 0.1451			
C allele	302 (52.8)	350 (61.8)	**χ^2^ = 9.50** ***p* = 0.0018**	Reference	
**A allele**	270 (47.2)	216 (38.2)		**1.45 (1.14–3.14)**	**0.002**
Dominant model					
A− carriers (CC)	85 (29.7)	114 (40.3)	**χ^2^ = 6.98** ***p* = 0.0083**	Reference	
**A+ carriers (AA + CA)**	201 (70.3)	169 (59.7)		**1.60 (1.13–2.26)**	**0.008**
***CDKN1B*** **rs34330**					
CC	188 (65.7)	205 (72.4)	χ^2^ = 4.84 *p* = 0.0888 *	Reference	
CT	84 (29.4)	72 (25.4)		1.27 (0.88–1.85)	0.2000
**TT**	14 (4.9)	6 (2.1)		**2.54 (0.96–6.76)**	**0.0500**
HWE	*p* = 0.2544	*p* = 0.9127			
C allele	460 (80.4)	482 (85.2)	**χ^2^ = 4.48** ***p* = 0.0343**	Reference	
**T allele**	112 (19.6)	84 (14.8)		**1.40 (1.02–1.91)**	**0.0300**
Dominant model					
T− carriers (CC)	188 (65.7)	205 (72.4)	χ^2^ = 2.99 *p* = 0.0837 *	Reference	
T+ carriers (TT + CT)	98 (34.3)	78 (27.6)		1.37 * (0.96–1.96)	0.08 *
***CDKN1B*** **rs2066827**					
TT	191 (66.8)	206 (72.8)	χ^2^ = 2.47 *p* = 0.2909	Reference	
TG	83 (29.0)	68 (24.0)		1.32 (0.90–1.92)	0.15
GG	12 (4.2)	9 (3.2)		1.44 (0.59–3.49)	0.42
HWE	*p* = 0.4386	*p* = 0.2552			
T allele	465 (81.3)	480 (84.8)	χ^2^ = 2.49 *p* = 0.1145	Reference	
G allele	107 (18.7)	86 (15.2)		1.28 (0.94–1.75)	0.11
Dominant model					
G− carriers (TT)	191 (66.8)	206 (72.8)	χ^2^ = 2.44 *p* = 0.1187	Reference	
G+ carriers (GG + TG)	95 (33.2)	77 (27.2)		1.33 (0.93–1.91)	0.12

HWE—Hardy–Weinberg equilibrium test; OR—odds ratio; 95%CI—95% confidence intervals. Statistically significant results are shown in bold. (*) indicates a trend.

**Table 3:** Genotype and allele frequencies of selected polymorphisms within the *CCND2* gene in CLL patients and the control group.

Polymorphic Site	CLL Patients n (%)	Controls n (%)	χ^2^ Test	OR (95%CI)	*p*-Value
***CCND2*** **rs3217933**					
TT	189 (66.1))	181 (64.0)	χ^2^ = 0.59 *p* = 0.7458	Reference	
TC	82 (28.7)	89 (31.4)		0.88 (0.61–1.27)	0.70
CC	15 (5.2)	13 (4.6)		1.11 (0.51–2.39)	0.80
HWE	*p* = 0.1297	*p* = 0.6287			
T allele	460 (80.4)	451 (79.9)	χ^2^ = 0.10 *p* = 0.7556	Reference	
C allele	112 (19.6)	115 (20.3)		0.95 (0.71–1.28)	0.76
Dominant model					
C− carriers (TT)	189 (66.1)	181 (64.0)	χ^2^ = 0.283 *p* = 0.5949	Reference	
C+ carriers (CC + TC)	97 (33.9)	102 (36.0)		0.91 (0.65–1.29)	0.59
***CCND2*** **rs3217901**					
AA	123 (43.0)	100 (35.3)	χ^2^ = 4.06 *p* = 0.1314	Reference	
**AG**	120 (42.0)	141 (49.8)		**0.69 (0.48–0.99)**	**0.04**
GG	43 (15.0)	42 (14.8)		1.20 (0.73–1.98)	0.47
HWE	*p* = 0.1297	*p* = 0.4993			
A allele	366 (64.0)	341 (60.2)	χ^2^ = 1.69 *p* = 0.1936	Reference	
G allele	206 (36.0)	225 (39.8)		0.85 (0.67–1.08)	0.19
Dominant model					
G− carriers (AA)	123 (43.0)	100 (35.3)	χ^2^ = 3.51 *p* = 0.0609 *	Reference	
G+ carriers (GG + AG)	163 (57.0)	183 (64.7)		0.72 * (0.52–1.02)	0.08 *
***CCND2*** **rs3217810**					
CC	214 (74.8)	233 (82.3)	χ^2^ = 5.37 *p* = 0.0683 *	Reference	
CT	63 (22.0)	46 (16.3)		1.49 * (0.98–2.28)	0.06 *
TT	9 (3.1)	4 (1.4)		2.45 (0.74–8.07)	0.22
HWE	*p* = 0.1122	*p* = 0.2552			
C allele	491 (85.8)	512 (90.5)	**χ^2^ = 5.81** ***p* = 0.0160**	Reference	
**T allele**	81 (14.2)	54 (9.5)		**1.56 (1.09–2.26)**	**0.02**
Dominant model					
T− carriers (CC)	214 (74.8)	233 (82.3)	**χ^2^ = 4.76** ***p* = 0.0291**	Reference	
**T+ carriers (TT + CT)**	72 (25.2)	50 (17.7)		**1.57 (1.05–2.35)**	**0.03**

HWE—Hardy–Weinberg equilibrium test; OR—odds ratio; 95%CI—95% confidence intervals. Statistically significant results are shown in bold. (*) indicates a trend.

**Table 4:** Haplotype frequencies of the investigated *CDKN1B* and *CCND2* gene polymorphisms among CLL patients and healthy subjects.

*CDKN1B* rs36228499	*CDKN1B* rs34330	*CDKN1B* rs2066827	*CCND2* rs3217933	*CCND2* rs3217901	*CCND2* rs3217810	CLL Patients	Controls	χ^2^ Test	OR (95%CI)	*p*-Value
**A**	**C**	**T**	**T**	**A**	**C**	**111.21 (0.194)**	**77.08 (0.136)**	**χ^2^ = 8.91** ***p* = 0.0029**	**1.65** **(1.19–2.29)**	**0.0029**
**C**	**C**	**G**	**T**	**A**	**C**	**26.38 (0.046)**	**2.68 (0.005)**	**χ^2^ = 20.51** ***p* = 0.000006**	**10.64** **(3.01–37.65)**	**0.000006**
C	C	T	C	G	C	14.22 (0.025)	25.12 (0.044)	χ^2^ = 2.95 *p*= 0.086 *	0.562 * (0.29–1.09)	0.0859 *
**C**	**C**	**T**	**T**	**A**	**C**	**104.04 (0.182)**	**142.55 (0.252)**	**χ^2^ = 7.50** ***p* = 0.006**	**0.66** **(0.49–0.89)**	**0.0062**
C	C	T	T	A	T	17.79 (0.031)	8.42 (0.015)	χ^2^ = 3.66 *p* = 0.0558 *	2.21 * (0.96–5.07)	0.0558 *
**C**	**C**	**T**	**T**	**G**	**C**	**29.95** **(0.052)**	**53.11** **(0.094)**	**χ^2^ = 6.67** ***p* = 0.0098**	**0.54** **(0.34–0.87)**	**0.0098**

χ^2^ global, df = 9 = 45.367 (frequency < 0.03 in all groups has been dropped), *p* = 0.0000008. OR—odds ratio; 95%CI—95% confidence intervals. Statistically significant results are shown in bold. (*) indicates a trend.

**Table 5:** Clinical significance of *CDKN1B* and *CCND2* gene polymorphic variants in CLL patients.

Genotype	Clinical Impact	
	**CLL Risk**	**CLL Course**
***CDKN1B*** **rs36228499**		
CC	-	Aggressive
AA + CA	Increased	Stable
***CDKN1B*** **rs34330**		
CC	-	Aggressive
CT + TT	Increased	Stable
***CDKN1B*** **rs2066827**		
TT	-	Aggressive
TG + GG	Increased *	Stable
***CCND2*** **rs3217933**		
TT	No	No
CC + TC	No	No
***CCND2*** **rs3217901**		
AA	-	Stable
AG	Decreased	Aggressive
GG	No	Stable
***CCND2*** **rs3217810**		
CC	-	Stable
CT + TT	Increased	Aggressive

(-) indicates the reference genotype, (*) indicates a trend.

**Table 6:** Association between *CDKN1B* gene polymorphisms and p27*^Kip1^* and cyclin D2 expression and apoptosis in CLL patients.

Polymorphic Site	CD19+CD5+ Cells		CD3+ Cells		Apoptotic Cells (%)	
	p27*^Kip1^*+	Cyclin D2+	p27*^Kip1^*+	Cyclin D2+	CD19+	CD3+
***CDKN1B*** **rs36228499**						
A− carriers (CC)	2880.50 (2627.00–3590.00)	204.50 (183.50–229.00)	3793.00 (3197.00–4938.00)	**245.00** **(237.00–256.00)**	4.35 (3.07–12.07)	11.34 (5.65–26.05)
A+ carriers (CA + AA)	3129.00 (2150.00–4263.00)	237.00 (204.00–288.00)	3807.00 (3039.00–4710.00)	**295.00** **(265.50–349.00)**	3.67 (2.48–8.34)	9.69 (3.84–15.88)
***p*** **-Value**	0.8610	0.0516 *	0.8696	**0.0027**	0.3141	0.4244
***CDKN1B*** **rs34330**						
T− carriers (CC)	3129.00 (1902,00–4142.00)	237.00 (204.00–288.00)	**3167.00** **(2722.00–4094.00)**	294.50 (263.00–322.00)	3.67 (2.44–5.87)	14.17 (4.89–17.10)
T+ carriers (CT + TT)	2780.50 (2469.50–4420.50)	218.50 (191.50–249.00)	**3921.00** **(3252.00–5240.00)**	266.00 (246.00–338.00)	3.95 (2.81–14.66)	8.94 (3.88–15.91)
***p*-Value**	0.4877	0.1360	**0.0105**	0.6620	0.1870	0.5773
***CDKN1B*** **rs2066827**						
G− carriers (TT)	3183.50 (2385.00–4183.50)	220.00 (192.00–272.00)	3802.50 (3069.50–4824.00)	268.50 (243.00–331.50)	4.07 (2.71–10.64)	11.36 (4.55–19.67)
G+ carriers (TG + GG)	2745.00 (2150.00–4616.00)	237.00 (207.00–271.00)	3579.00 (2926.00–4510.00)	269.00 (261.00–307.00)	3.29 (2.43–7.34)	10.52 (3.44–15.88)
***p*** **-Value**	0.5286	0.6603	0.7612	0.8337	0.2373	0.5212

Median proportions and interquartile ranges (25th and 75th interquartiles) are presented. Differences between the studied groups were evaluated using the non-parametric Mann–Whitney U test. Significant results shown in bold. (*) indicates a trend.

## Data Availability

The datasets generated and/or analyzed during the current study are available from the corresponding author upon reasonable request.

## Supplementary Materials

The following supporting information can be downloaded at: https://www.mdpi.com/article/10.3390/ijms252111705/s1.
