# Supplementary material for: Association of Genetic Variants at the CDKN1B and CCND2 Loci Encoding p27Kip1 and Cyclin D2 Cell Cycle Regulators with Susceptibility and Clinical Course of Chronic Lymphocytic Leukemia
Source: Int J Mol Sci. 2024 Oct 31;25(21):11705. doi: 10.3390/ijms252111705 (PMC11546115; doi:10.3390/ijms252111705)
Supplement: Supplementary file 1 [file ijms-25-11705-s001.zip › ijms-3265975-supplementary.pdf]

SUPPLEMENTARY MATERIALS

Materials and Methods

DNA genotyping

Genotyping of the *CDKN1B* (p27^{Kip1}) variants: rs36228499 (c.-838C>A) located in the promotor region of *CDKN1B*, and rs2066827 (c.326T>G), located in exon 1 as well as within the *CCND2* (cyclin D2) gene: two intronic tagSNPS: rs3217810 (g.4279105C>T), rs3217901 (g.4405389A>G) and rs3217933 (c.*3825T>C) located in the 3′UTR region of *CCND2*, were genotyped with the allelic discrimination (AD) technique with use of the appropriate validated and predesigned TaqMan®SNP Genotyping Assays (C__86361295_10, C__11916245_10 (Catalog #4351379), C__25605613_10, C_11333084_10 (Catalog #4351379), and C_27487726_10) (Thermo Fisher Scientific, Life Technologies, Poland). The TaqMan®SNP Genotyping Assay was controlled (25% of randomly chosen samples from both groups) to check for genotyping accuracy. Identical genotypes were identified in all repeated samples. Identification of the sample subjects during genotyping was blinded.

The SNP was genotyped with the use of the PCR-RFLP method using the specific restriction enzyme AccII (BSH1236I; BstUI; MvnI). The 599-bp promoter region of the *CDKN1B* gene, encompassing the c.-79C>T (rs34330) polymorphism site, was amplified using a pair of primers: sense 5′-GCCATATTGGGCCACTAAAA-3′ and antisense 5′-TCTCTGCAGTGCTTCTCCAA-3′. The PCR-digested products generated allele-specific digestion fragments: 9, 127, 145, 89, and 229 bp for the C allele and 9, 127, 145, 318 bp for the T allele. The primers were designed according to the complete *CDKN1B* (NC_000012.12) sequence derived from the NCBI Sequence Viewer (http://www.ncbi.nlm.nih.gov/) with use of the free Primer3 (v. 0.4.0) software (http://bioinfo.ut.ee/primer3-0.4.0/) [91,92].

To confirm the results of the PCR-RFLP analysis, 20% of the samples were genotyped twice and interpretation of the results was performed by two different observers; the results conformed with the original analysis.

Immunostaining of p27^{Kip1} protein and cyclin D2, and flow cytometric analysis

Cryopreserved PBMCs from CLL patients were thawed, washed twice in PBS, and divided into tubes at a concentration of 5 x 10^5 cells per tube. For detection of p27^{Kip1} protein, the cells were incubated with PerCP anti-human CD19 (Catalog #345778, BD Biosciences, San Diego, USA), PE anti-human CD5 (Catalog #345782, BD Biosciences, San Diego, USA) or PE anti- human CD3 (Catalog #345765, BD Biosciences, San Diego, USA) monoclonal primary antibodies (mAbs). Next, the cells were fixed, permeabilized, and stained with mAbs to p27^{Kip1} (Catalog #554069, Pharmingen, BD Biosciences, San Diego, USA) according to the method described by Wolowiec et al. [29]. As a secondary fluorochrome-labeled antibody with specificity for the primary antibody to p27^{Kip1}, FITC rat anti-mouse IgG1 (Catalog #553443, Pharmingen, BD Biosciences, San Diego, USA) was used. For detection of cyclin D2, the cells were incubated with PerCP anti-human CD19, PE anti-human CD5 or PE anti-human CD3 mAbs, fixed, permeabilized, and stained with an FITC anti-human cyclin D2 antibody (Catalog #sc-53637, Santa Cruz Biotechnology, Inc, Heidelberg, Germany) according to the method described by Gong et al. [93] and Wolowiec et al. [29]. Appropriate fluorochrome-labeled isotype control antibodies from BD Biosciences (BD Biosciences, San Diego, CA, USA) and Santa Cruz Biotechnology (Santa Cruz Biotechnology, Inc, Heidelberg, Germany) were used to confirm expression specificity and for gate settings in each case (PerCP mouse IgG1: Catalog #349044, PE mouse IgG2a: Catalog #555574, PE mouse IgG1: Catalog #555749, FITC mouse IgG1: Catalog #555748, FITC mouse IgG2b: Catalog sc-2857). At least 50,000 events per sample were analyzed. In order to

demonstrate quantitative expression of the studied molecules at the single-cell level, the results are shown as mean fluorescence intensity (MFI) values and expressed in arbitrary units (AU). FACS Diva software 8.0 (Becton Dickinson, BD Biosciences, San Diego, USA) was used for statistical analysis of the acquired data.

Determination of apoptosis (assessed as mitochondrial membrane potential [ΔΨm low] changes)

The MitoTracker Red CMXRos probe is a red fluorescent dye that is passively transported through the cell membrane and directly assembled in active mitochondria. The accumulation of this dye depends on the mitochondrial membrane potential. Since apoptotic cells lose their mitochondrial membrane potential [94], the CMXRos staining of mitochondria decreases (CMXRos low). The CMXRos was prepared as a stock solution in DMSO, according to the manufacturer's instructions. PBMCs from CLL patients were resuspended in 0.5 ml of complete culture medium with 0.2 μM CMXRos working solution and incubated for 30 min at 37°C. For the last 15 min of incubation, FITC anti-human CD19 or FITC anti-human CD3 monoclonal antibodies (Catalog #345776 or Catalog #345764, respectively; BD Biosciences, San Diego, USA) were added. As appropriate fluorochrome-labeled isotype control antibodies, FITC mouse IgG1: Catalog #555748 (Pharmingen, BD Biosciences, San Diego, USA) was used. At least 50,000 events per sample were acquired and analyzed by a flow cytometer immediately after the incubation period. The results are expressed as the percentages of apoptotic cells within the CD19+ and CD3+ cell subpopulations.

Table S1. Distribution of genotypes for selected *CDKN1B* and *CCND2* polymorphisms in a group of 47 CLL patients.

Polymorphic site	Observed group size (n)	Observed frequency (%)	Expected group size (n)	Expected frequency (%)	HWE (*p*-Value)	χ^2 test
***CDKN1B*rs36228499**						
CC	18	38.30	15.51	33.00		
CA	18	38.30	22.98	48.89	0.1374	$\chi^2 = 2.2064$
AA	11	23.40	8.51	18.11		$p = 0.3318$
***CDKN1B*rs34330**						
CC	21	46.67	21.36	47.46		
CT	20	44.44	19.29	42.86	0.8047	$\chi^2 = 0.0081$
TT	4	8.89	4.36	9.68		$p = 0.9960$
***CDKN1B*rs3206827**						
TT	28	60.87	27.40	59.56		
TG	15	32.61	16.21	35.23	0.6136	$\chi^2 = 0.0356$
GG	3	6.52	2.40	5.21		$p = 0.9823$
***CCND2*rs3217933**						
**TT**	**26**	**57.78**	**21.36**	**47.46**		
**TC**	**10**	**22.22**	**19.29**	**42.86**	**0.0012**	$\chi^2 = 10.4358$
**CC**	**9**	**20.00**	**4.36**	**9.68**		$p = 0.0054$
***CCND2*rs3217901**						
AA	21	45.65	18.92	41.13		
AG	17	36.96	21.16	46.01	0.1821	$\chi^2 = 1.78$
GG	8	17.39	5.92	12.87		$p = 0.4107$
***CCND2*rs3217810**						
CC	31	65.96	30.72	65.37		
CT	14	29.79	14.55	30.96	0.7944	$\chi^2 = 0.0308$
TT	2	4.26	1.72	3.67		$p = 0.9847$

HWE–Hardy-Weinberg equilibrium test; OR–Odds Ratio; 95%CI–95% Confidence Intervals. Significant results are shown in bold.

Table S2. Association between *CDKN1B* and *CCND2* polymorphisms and IgVH mutation status

**Polymorphic site**	**IgVH status (n)**		**χ^2 test**
	**Unmutated**	**Mutated**	
***CDKN1B*rs36228499**			
CC	8	8	$\chi^2 = 1.0636$
CA	6	10	$p = 0.5876$
AA	2	5	
***CDKN1B*rs34330**			
CC	9	8	$\chi^2 = 1.2092$
CT	6	11	$p = 0.5463$
TT	1	2	
***CDKN1B*rs3206827**			
TT	9	14	$\chi^2 = 0.8269$
TG	5	7	$p = 0.6614$
GG	2	1	
***CCND2*rs3217933**			
**TT**	10	11	$\chi^2 = 0.7831$
**TC**	4	5	$p = 0.6760$
**CC**	2	5	
***CCND2*rs3217901**			
AA	8	9	$\chi^2 = 1.7763$
AG	7	9	$p = 0.4114$
GG	1	5	
***CCND2*rs3217810**			
CC	11	16	$\chi^2 = 0.7857$
CT	5	6	$p = 0.6751$
TT	0	1	

Table S3. Laboratory characteristics of CLL patients by *CDKN1B* genotype.

Polymorphic site	WBC count ($1x10^9$/l)	Lymphocyte count ($1x10^9$/l)	Hb level (g/dl)	Platelet count ($1x10^9$/l)	LDH (U/l)	β2-microglobulin (mg/l)
CDKN1B**rs36228499**						
A- carriers (CC)	46.28 ± 39.28	38.99 ± 35.95	12.66 ± 2.02	142.64 ± 44.82	205.60 ± 79.07	3.32 ± 2.13
A+ carriers (CA + AA)	49.36 ± 32.01	42.22 ± 30.37	13.09 ± 1.66	155.96 ± 57.60	201.69 ± 72.81	3.37 ± 1.07
p**-Value**	0.5999	0.6606	0.4756	0.4579	0.9191	0.9461
CDKN1B**rs34330**						
T- carriers (CC)	51.98 ± 40.28	43.44 ± 37.89	12.96 ± 1.77	152.06 ± 61.70	208.30 ± 87.45	3.21 ± 1.19
T+ carriers (CT + TT)	45.54 ± 29.69	39.41 ± 28.08	12.93 ± 1.83	150.74 ± 47.55	197.45 ± 59.27	3.43 ± 1.76
p**-Value**	0.3794	0.5484	0.1366	0.6257	0.2528	0.1298
CDKN1B **rs2066827**						
G- carriers (TT)	54.67 ± 36.91	45.87 ± 34.80	13.26 ± 1.75	143.83 ± 45.27	200.82 ± 48.23	3.27 ± 1.79
G+ carriers (TG + GG)	38.70 ± 28.67	34.16 ± 27.18	12.46 ± 1.77	162.50 ± 63.34	204.60 ± 95.01	3.47 ± 1.22
p**-Value**	0.0921*	0.2716	0.1668	0.2833	0.9083	0.7706

The mean values and standard deviation (SD) are presented. Differences between the studied groups were evaluated using the non-parametric Mann-Whitney U test. (*) means a trend.

Table S4. Laboratory characteristics of CLL patients by *CCND2* genotype.

Polymorphic site	WBC count ($1x10^9/l$)	Lymphocyte count ($1x10^9/l$)	Hb level (g/dl)	Platelet count ($1x10^9/l$)	LDH (U/l)	β2-microglobulin (mg/l)
***CCND2*rs3217933**						
TT	48.25 ± 37.46	39.66 ± 34.98	13.11 ± 1.96	158.19 ± 48.07	197.14 ± 84.77	2.90 ± 1.41
TC	37.29 ± 21.19	31.97 ± 20.43	13.12 ± 0.68	131.44 ± 39.96	238.00 ± 53.68	4.67 ± 1.75
CC	51.92 ± 32.51	46.28 ± 30.51	12.20 ± 2.07	163.67 ± 70.53	195.25 ± 18.41	3.27 ± 0.63
***p*-Value**						
TT vs TC	0.8979	0.8890	0.9999	0.4480	0.6956	0.0851*
TT vs CC	0.9889	0.9223	0.9103	0.9662	0.9990	0.9199
TC vs CC	0.6999	0.7770	0.8999	0.4352	0.7580	0.4266
***CCND2*rs3217901**						
AA	49.66 ± 35.56	42.79 ± 33.49	12.94 ± 1.82	153.88 ± 46.25	188.64 ± 66.67	3.27 ± 1.52
AG	49.61 ± 38.05	40.30 ± 35.33	13.18 ± 1.73	122.93 ± 50.81	243.40 ± 109.55	3.47 ± 1.97
GG	43.60 ± 28.39	38.89 ± 27.39	12.58 ± 1.93	175.00 ± 64.22	192.60 ± 17.00	3.27 ± 0.51
***p*-Value**						
AA vs AG	0.9999	0.9888	0.9370	0.5450	0.9768	0.9679
AA vs GG	0.9789	0.8999	0.9705	0.7946	0.9999	0.9916
AG vs GG	0.8999	0.9770	0.8691	0.3067	0.8998	0.9987
***CCND2* rs3217810**						
T- carriers (CC)	42.55 ± 29.17	36.02 ± 27.05	13.23 ± 1.89	148.23 ± 43.84	217.08 ± 78.51	2.93 ± 1.30
T+ carriers (CT + TT)	58.93 ± 41.18	50.07 ± 38.92	12.41 ± 1.47	157.00 ± 69.03	179.13 ± 57.93	3.96 ± 1.75
***p*-Value**	0.9210	0.8130	0.8651	0.9656	0.7409	0.8582

The mean values and standard deviation (SD) were presented. Differences between the studied groups were evaluated using the non-parametric Kruskal-Wallis one-way ANOVA or Mann-Whitney U test. (*) means a trend.

Table S5. Association between *CCND2* gene polymorphisms and p27^{Kip1} and cyclin D2 expression and the apoptosis in CLL patient.

**Polymorphic site**	**CD19+CD5+ cells**		**CD3+ cells**		**Apoptotic cells (%)**	
	**p27^{Kip1}+**	**Cyclin D2+**	**p27^{Kip1}+**	**Cyclin D2+**	**CD19+**	**CD3+**
***CCND2*rs3217933**						
TT	3,048.50 (2,344.00 – 4,142.00)	237.50 (225.00 – 280.00)	3,558.00 (3,047.00 – 4,710.00)	307.00 (247.00 – 373.00)	3.95 (3.07 – 8.39)	13.65 (3.91 – 22.24)
TC	3,313.00 (2,694.00 – 5,379.00)	216.50 (192.00 – 288.00)	3,826.00 (3,039.00 – 5,621.00)	261.50 (247.50 – 276.00)	5.59 (2.48 – 15.09)	10.94 (4.89 – 16.18)
CC	2,360.00 (1,902.00 – 5,379.00)	196.00 (178.00 – 288.00)	3,807.00 (2,926.00 – 3,952.00)	263.50 (230.50 – 278.50)	3.29 (2.44 – 15.09)	4.60 (2.81 – 11.98)
***p*-Value**						
TT vs TC	0.9998	0.6448	0.9876	0.3646	0.8997	0.9879
TT vs CC	0.4733	0.2718	0.9765	0.2513	0.9998	0.3214
TC vs CC	0.2595	0.8671	0.9999	0.9889	0.8797	0.7959
***CCND2*rs3217901**						
AA	2,768.00 (1,975.00 – 3,585.00)	237.00 (213.50 – 269.00)	3,821.00 (3,092.00 – 4,938.00)	270.00 (247.00 – 396.00)	3.65 (2.55 – 5.19)	9.06 (3.84 – 26.05)
AG	3,503.00 (3,129.00 – 4,225.00)	260.00 (192.00 – 288.00)	3,840.00 (3,197.00 – 4,998.00)	284.00 (242.00 – 338.00)	5.87 (2.90 – 8.39)	14.17 (5.65 – 16.90)
GG	2,255.00 (1,812.50 – 3,669.00)	190.00 (178.00 – 204.00)	3,487.00 (2,980.00 – 4,152.00)	268.00 (219.00 – 285.00)	5.14 (2.09 – 11.72)	9.08 (3.13 – 14.23)
***p*-Value**						
AA vs AG	0.3222	0.9989	0.9999	0.9980	0.9660	0.9680
AA vs GG	0.9870	0.0872*	0.9899	0.6600	0.9960	0.9789
AG vs GG	0.1228	0.0880*	0.9850	0.9980	0.9960	0.9987
***CCND2*rs3217810**						
T- carriers (CC)	2,768.00 (1,914.00 – 3,503.00)	235.50 (204.00 – 288.00)	3,890.00 (3,045.00 – 4,938.00)	271.00 (252.50 – 349.00)	3.82 (2.55 – 9.77)	13.07 (4.50 – 19.50)
T+ carriers (CT + TT)	3,582.50 (2,603.50 – 4,704.50)	227.00 (188.00 – 263.00)	3,530.00 (3,009.00 – 4,327.50)	263.00 (242.00 – 320.00)	3.68 (2.40 – 8.37)	7.92 (3.64 – 15.15)
***p*-Value**	0.5286	0.3399	0.7612	0.4518	0.2373	0.5212

Median proportions and interquartile ranges (25th and 75th interquartiles) are presented. Differences between studied groups were evaluated using the non-parametric Kruskal-Wallis one-way ANOVA or Mann-Whitney U test. (*) means a trend.

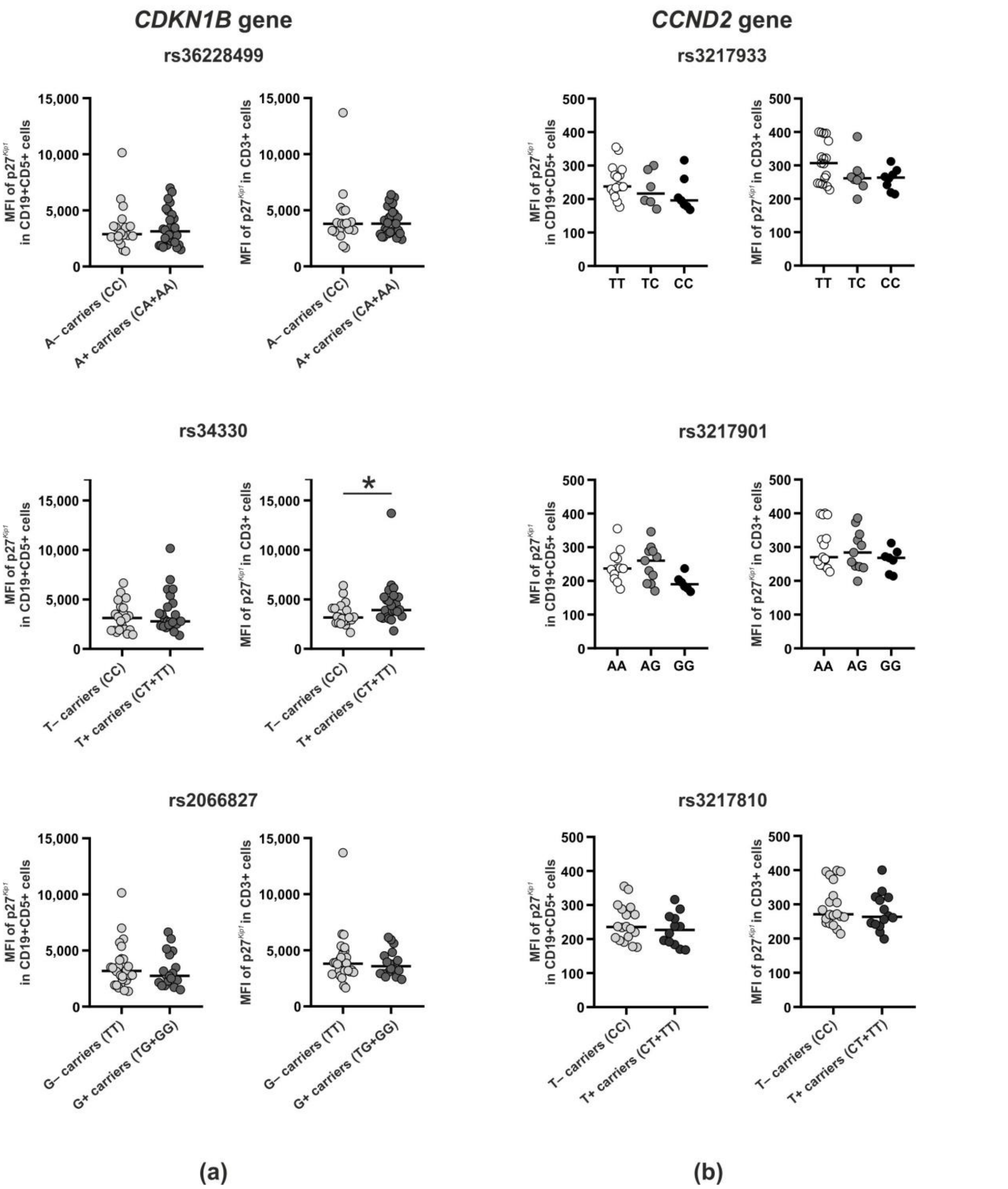

Figure S1. Association between genetic variants of the *CDKN1B* and CCND2 genes and expression of the p27^{Kip1} and cyclin D2 proteins in CLL patients. **Panel a**: The graphs show the mean fluorescence intensity (MFI) of p27^{Kip1} protein in PB CD19+CD5+ and CD3+ cells in CLL patients divided according to the genetic variants of the *CDKN1B*rs36228499, *CDKN1B*rs34330, and *CDKN1B*rs2066827 polymorphic sites. **Panel b**: The graphs show the MFI of cyclin D2 protein in PB CD19+CD5+ and CD3+ cells in CLL patients divided according to the genetic variants of the *CCND2*rs3217933, *CCND2*rs3217901, and *CCND2*rs3217810 polymorphic sites. The horizontal lines represent the median values. Differences between studied groups were evaluated using the non-parametric Kruskal-Wallis one-way ANOVA or Mann-Whitney U test. The horizontal lines represent the median values. Differences between studied groups were evaluated using the Mann-Whitney U test. (*) signifies a statistically significant difference $p < 0.05$.

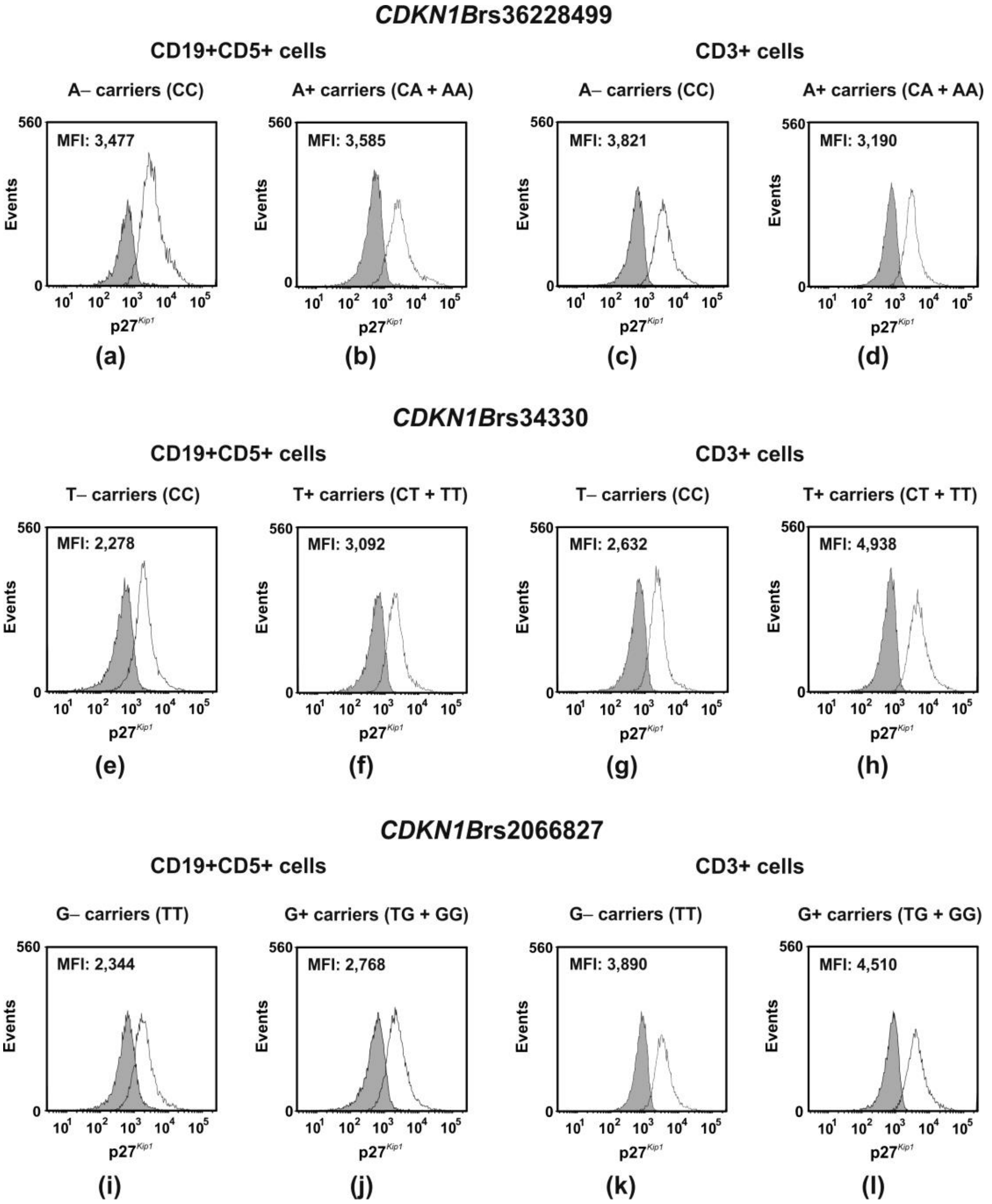

Figure S2. Cytometric analysis of p27^{Kip1} protein expression in CLL patients. Histograms show cytometric analysis of p27^{Kip1} protein expression in PB CD19+CD5+ (**a**, **b**, **e**, **f**, **i**, **j**) and CD3+ (**c**, **d**, **g**, **h**, **k**, **l**) cells co-expressing p27^{Kip1} protein in CLL patients divided according to the genetic variants of the *CDKN1B*rs36228499 (**a–d**), *CDKN1B*rs34330 (**e–h**), and *CDKN1B*rs2066827 (**i–l**) polymorphic sites. PBMCs were gated using FSC/SSC profiles (R1) – not shown, followed by gating on CD19+CD5+ (**a**, **b**, **e**, **f**, **i**, **j**) or CD3+ (**c**, **d**, **g**, **h**, **k**, **l**) (R2 – not shown) to identify CD19+CD5+ and CD3+ cells for further analysis of p27^{Kip1} protein expression in PB CD19+CD5+ and CD3+ cells. Black line curves show p27^{Kip1}-fluorescence of cells within PB CD19+CD5+ and CD3+ cells. Gray areas represent the isotype controls. The numbers located on the histograms represent the p27^{Kip1}-dependent signal intensity (MFI).
